# Cytogenomics of *Myloplus tiete* reveals conserved satellite DNAs since the Late Eocene in Serrasalmidae (Teleostei, Characiformes)

**DOI:** 10.1007/s10577-026-09801-w

**Published:** 2026-05-08

**Authors:** Francisco de Menezes Cavalcante Sassi, Giovanna Igepi, Beatriz Lopes Torgan, Caio Augusto Gomes Goes, Rodrigo Zeni dos Santos, Maria Eduarda Gisloti-Ribeiro, Diogo Teruo Hashimoto, Fábio Porto-Foresti, Ricardo Utsunomia

**Affiliations:** 1 MOE Key Laboratory of Freshwater Fish Reproduction and Development, School of Life Sciences, Southwest University, Chongqing, 400715 China; 2 Faculty of Sciences, São Paulo State University (UNESP), Bauru, SP 17033-360 Brazil; 3 Aquaculture Center of UNESP, São Paulo State University, Jaboticabal, SP 14884-900 Brazil

**Keywords:** Karyotype, Satellitome, Consensus Turnover Rate, Neotropical Fish, Concerted Evolution

## Abstract

The Neotropical family Serrasalmidae (pacus and piranhas) exhibits remarkable karyotype conservation, yet cytogenetic data remain limited for several genera, particularly *Myloplus*. Here we present the first integrative cytogenomic characterization of *Myloplus tiete*, an endemic and near-threatened species from the upper Paraná River basin, combining classical cytogenetics with satellitome analysis. Karyotyping of 12 specimens collected from Rio Grande, Frutal—MG, Brazil, revealed a consistent diploid number of 2n = 58 chromosomes with karyotype formula 16m + 20sm + 22a, while C-banding identified heterochromatic blocks predominantly in pericentromeric and subtelomeric regions. Genome sequencing and iterative satDNA mining identified 32 satellite DNA families, with repeat lengths ranging from 24 to 2,265 bp and a predominance of AT-rich sequences. Comparative analysis with the Colossomatinae species *Colossoma macropomum* and *Piaractus mesopotamicus* revealed 12 conserved satDNAs across ~ 40 million years of divergence, with moderate consensus turnover rates. FISH mapping of the ten most abundant satDNAs revealed diverse chromosomal distributions, including pericentromeric, subtelomeric, and dispersed patterns, highlighting the diverse genomic integration of conserved repeats. These findings provide evidence for long-term evolutionary conservation of both karyotype architecture and satDNA repertoires across Serrasalmidae subfamilies and suggest that structural or functional constraints may contribute to shaping the evolution of repetitive DNA in this ecologically and aquaculturally important fish group.

**Supplementary Information:**

The online version contains supplementary material available at 10.1007/s10577-026-09801-w.

## Introduction

Satellite DNAs (satDNAs) are highly abundant repetitive sequences organized in tandem arrays, typically concentrated and are the main components of heterochromatic regions, although euchromatic occurrences have also been reported (Garrido-Ramos 2017). Beyond their structural abundance, these sequences play fundamental roles in chromosome dynamics, including kinetochore assembly and the organization of centromeres and telomeres (Shapiro et al. 2005; Louzada et al. 2020; Talbert and Henikoff 2022). SatDNAs evolve primarily through concerted evolution mechanisms and are among the fastest-evolving components of eukaryotic genomes (Garrido-Ramos 2017; Šatović-Vukšić and Plohl 2023), leading to the homogenization of sequence variants within reproductively isolated populations (Plohl et al. 2012). In recent years, advances in bioinformatics have enabled the comprehensive characterization of satDNA repertoires, providing new insights into the evolutionary dynamics, turnover, and genomic organization of repetitive sequences across diverse taxonomic groups, including teleost fishes (Ruiz-Ruano et al. 2016).

The Neotropical region, encompassing tropical areas of South and Central America (Morrone et al. 2018), harbors the world's most representative continental ichthyofauna, comprising approximately 6,300 described species (Fricke et al. 2026). Among the most diverse orders, Characiformes stands out for its extensive phenotypic diversity (Burns and Sidlauskas 2019) and accelerated speciation processes, particularly within the families Anostomidae, Characidae, and Serrasalmidae, which collectively represent 68% of the group's diversity (Melo et al. 2022). Commonly known as pacus and piranhas, Serrasalmidae includes 16 genera with more than 100 valid species (Fricke et al. 2026). This family is organized into three well-supported subfamilies: Myleinae, Serrasalminae, and Colossomatinae (Mateussi et al. 2020; Kolmann et al. 2021). These fishes are distributed throughout tropical and subtropical South America and exhibit diverse trophic ecologies (Melo et al. 2022), including carnivory, omnivory, herbivory, and lepidophagy (Mateussi et al. 2020; Kolmann et al. 2021). Beyond their ecological significance, Serrasalmidae represents the major native fish group in South American aquaculture (Valladão et al. 2018) and plays crucial ecological roles in forest structuring through long-distance seed dispersal (Correa et al. 2015, 2016).

Cytogenetic studies have revealed substantial karyotype conservation within Serrasalmidae subfamilies. The Myleinae subfamily consistently exhibits 2n = 58 chromosomes (Adriano et al. 2006), while Colossomatinae species possess 2n = 54 chromosomes (Cestari et al. 1992; Favarato et al. 2021; Nakayama et al. 2002), and Serrasalminae shows variation ranging from 2n = 58–64 (Favarato et al. 2021; Nirchio et al. 2003; Ribeiro et al. 2014). Recent advances in molecular cytogenetics have enabled detailed characterization of repetitive DNA elements, particularly satDNAs, in Colossomatinae species. Comprehensive satellitome catalogs for *Piaractus mesopotamicus* and *Colossoma macropomum* have revealed not only a remarkable conservation of the chromosomal distribution of repetitive sequences spanning more than 30 million years (My), but also a high similarity in their overall satDNA repertoires (Goes et al. 2023).

However, cytogenetic data remain scarce for certain groups, especially the genus *Myloplus* and other Myleinae representatives. Despite being the third most species-rich genus within Serrasalmidae (Fricke et al. 2026), only three species have known karyotypes in *Myloplus*: *M. rubripinnis*, *M. asterias*, and *M. schomburgkii*, all from the Amazonian region and exhibiting 2n = 58 chromosomes (Favarato et al. 2021). *Myloplus tiete* is a near-threatened species (Brejão et al. 2024) originally distributed throughout the Paraná-Paraguay river basin, but with decreasing population sizes, lacks cytogenetic characterization, thus presenting an important opportunity to expand our understanding of chromosomal evolution and repetitive DNA dynamics within this group. Therefore, our study presents the first comprehensive cytogenomic characterization of *M. tiete*, including karyotype description and satellitome analysis. We also conducted comparative analyses with satellitomes of *P. mesopotamicus* and *C. macropomum*, representing the Colossomatinae subfamily. Our results support the conservative tendency of both chromosomal numbers and satDNA catalogs in Serrasalmidae and provide a cytogenomic framework for understanding chromosomal and repetitive DNA evolution in the family.

## Material and Methods

### Sampling and cytogenetic analysis

**Fig. 1:**
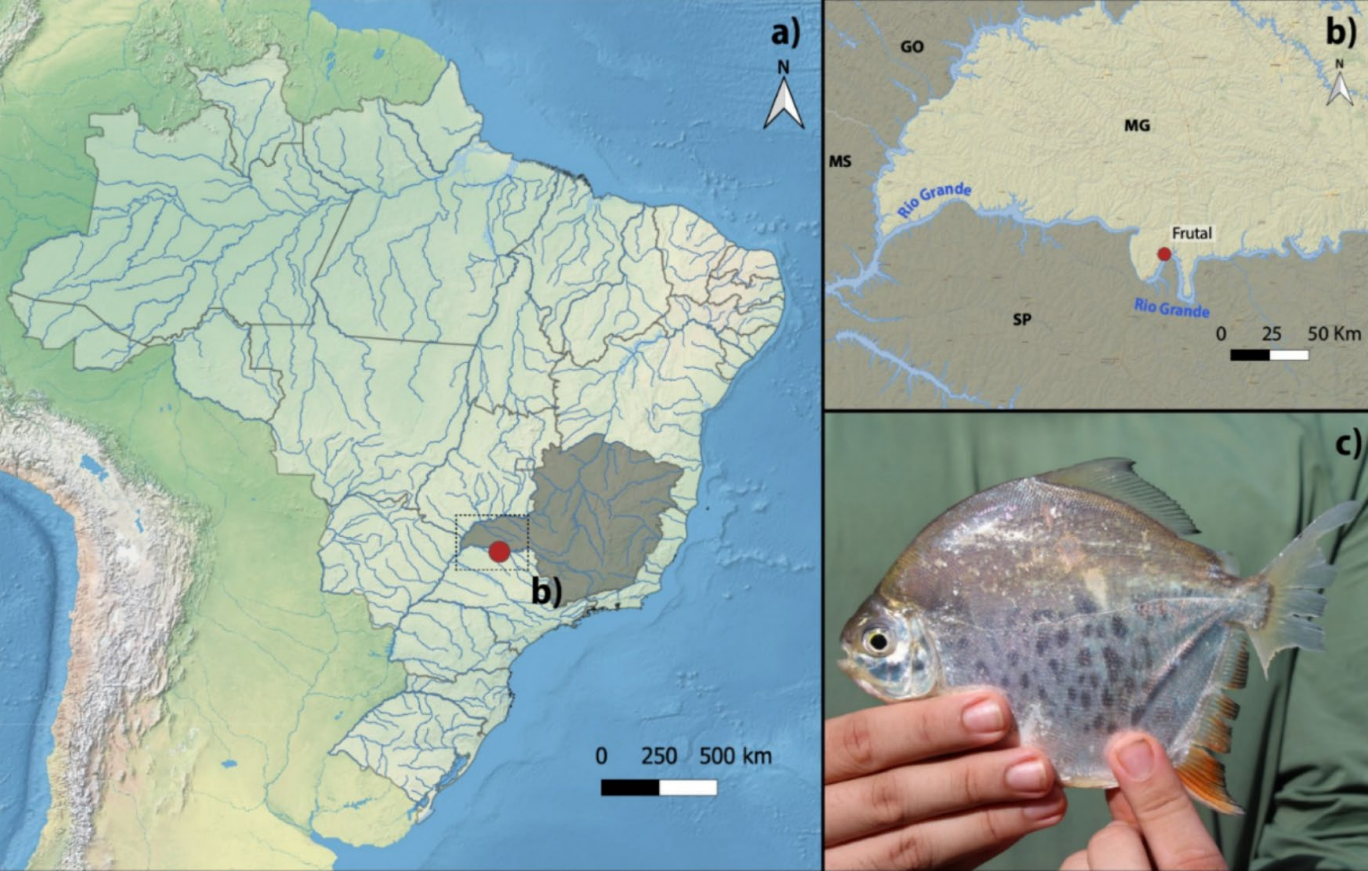
**A)** Map of South America, with the Brazilian political territory delimited; **b)** a zoom into the sampling state (MG), with the red dot indicating the sampling site of *Myloplus tiete* at Rio Grande, Frutal – MG**; c)** an exemplar of the species. Photo by Douglas Lopes © (via iNaturalist), some rights reserved (CC-BY)

We collected 12 individuals (6 males and 6 females) of *M. tiete* from the population of Rio Grande (Fig. 1), in the city of Frutal—MG, Brazil (20°08′48.1’’S; 49°04′17.0’’W). All specimens were collected using nets and were immediately stored in aerated containers. The procedures of collection, maintenance, and animal analysis followed the international standards for animal experimentation and were approved by the Ethics Committee on Animal Use of the São Paulo State University, under protocol 1227. Collection was authorized by SISBIO (permit no. 97277–1).

Mitotic chromosomes were obtained following the protocol established by Foresti et al. (1981). Heterochromatin bands were obtained via the classical C-banding technique (Sumner 1972). All images were captured using cellSens Standard 1.14 (Olympus), with a digital camera Olympus Qcolor5 on a fluorescence microscope (BX50, Olympus).

### DNA extraction and genome sequencing

Total genomic DNA was obtained from the liver tissue of one female individual of *M. tiete* using NucleoSpin® Tissue Kit (Quiagen), following the manufacturer’s instructions. The integrity of the gDNA was checked in 1% agarose gel. Genomic DNA sequencing was performed on the BGISEQ-500 platform at BGI (BGI Shenzhen Corporation, Shenzhen, China), generating 2 × 150 bp paired-end reads and a total of 2.42 Gb of raw data. The raw reads were deposited in the Sequence Read Archive (SRA) under accession number SRR37277025.

### Satellitome characterization and bioinformatic analysis

The first step to characterize the satellitome of *M. tiete* was to quality-check and trim the sequencing library with Trimmomatic (Bolger et al. 2014). A subset of 2 × 500,000 reads was randomly selected and used as input for TAREAN (Novák et al. 2020). The discovered satDNAs were removed from the original library using the software DeconSeq (Schmieder and Edwards 2011), and a new subsample of 2 × 500,000 reads was created and analyzed with TAREAN. These steps were iterated until no new satDNAs were found.

Other repetitive sequences, such as multigene families and transposable elements, were removed from the dataset before a homology search with RepeatMasker (Smit et al. 2020) to eliminate possible redundancies in the putative satDNAs and to group the sequences into the same variant (SV), variants of the same satDNA (V) or superfamilies (SF), based on the similarity between two consensus sequences (> 95%, > 80% and > 50%, respectively), following previously established studies (Ruiz-Ruano et al. 2016). All new satDNAs were deposited on GenBank, under PZ024035—PZ024066 accession numbers.

We utilized 2 × 5,000,000 reads to calculate the abundance and divergence of each new satDNA using RepeatMasker software (Smit et al. 2020) and a custom Python script (https://github.com/fjruizruano/ngsprotocols/blob/master/repeat_masker_run_big.py). Abundance is calculated through the division of mapped reads by the number of nucleotides analyzed. In addition, the script calcDivergenceFromAlign.py in Repeat Masker was used to estimate the divergence of each satDNA, using the Kimura-2-parameter model. The final catalog of satDNAs was named following the guidelines of Ruiz-Ruano et al. (2016), using the term “MtiSat” followed by the catalog number in decreasing order of abundance and the number of base pairs of each monomer.

Considering the previously described conservation of satDNA sequences between Colossomatinae species (Goes et al. 2023), we compared the satellitome of *M. tiete* with those of *C. macropomum* and *P. mesopotamicus* (Goes et al. 2023). For this, RepeatMasker was applied to detect similarities between the consensus sequences of the three catalogs with a custom script (https://github.com/fjruizruano/ngs-protocols/blob/master/rm_homology.py). SatDNA sequences of the three species were manually aligned, and those with at least 50% similarity were considered conserved. Pairwise genetic distances were calculated using the Kimura 2-parameter (K2P) method through a custom script in R (R Core Team 2019) using the packages ape 5.0 (Paradis and Schliep 2019) and Biostrings (Pagès et al. 2025). For satDNAs with high divergence, the Jukes-Cantor correction was applied to reduce potential infinite distances estimates when substitution saturation occurred. Consensus turnover rates (CTR) were calculated as CTR = K/2 T, where K represents the K2P distance and T is the divergence time in years. Based on mean estimates from TimeTree5 (Kumar et al. 2022), species divergence times were set at 40 Mya for *M. tiete* versus both *C. macropomum* and *P. mesopotamicus*, and 34.9 Mya for *C. macropomum* versus *P. mesopotamicus*. Statistical analyses and visualizations were performed in R using tidyverse (Wickham et al. 2019).

### Primer design and Fluorescence in situ Hybridization (FISH)

We selected the ten most abundant satDNAs from the *M. tiete* catalog for fluorescence in situ hybridization (FISH). For this, primers (**Supplementary Table 1**) were manually designed and checked for the formation of self-annealing or hairpins with the OligoCalc (Biotools) and Multiple Primer Analyzer (Thermofisher). Probes were constructed using digoxigenin-11-dUTP in PCR reactions using the obtained *M. tiete* total DNA as template.

FISH experiments followed the protocol established by Pinkel et al. (1986), with some adaptations. Briefly, the chromosomes were treated with 0.005% Pepsin/10 mM HCl for 5 min and fixed in 1% formaldehyde in 1 × PBS/50 mM MgCl_2_. After dehydration in ethanol series (70%, 80% and 100%) for 3 min each, chromosomes were denatured in 70% formamide/2 × SSC for 2 min at 70º C. The slides were washed in 0.2 × SSC/15% formamide at 42 °C, followed by washes in 0.1 × SSC for 15 min at 60 °C post-hybridization, and probe detection was performed with anti-digoxigenin-rhodamine. The chromosomes were counterstained with DAPI (4′,6-diamino-2-phenylindole, Vector Laboratories, Burlingame, CA, USA), and images were captured using an optical microscope (Olympus BX61) with DP Control software (Olympus®, Hamburg, Germany).

## Results

### First description of *M. tiete* karyotype

**Fig. 2:**
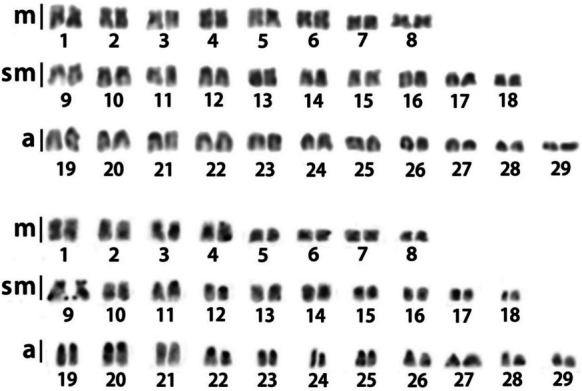
Karyotype of *Myloplus tiete* after Giemsa staining (upper) and C-band (lower)

All individuals presented 2n = 58 chromosomes, with a karyotype formula of 16m + 20sm + 22a (Fig. 2). No sexual polymorphism (heteromorphic sex chromosomes) or extra chromosomes were identified. Heterochromatic blocks were identified predominantly in centromeres of acrocentric chromosomes, in addition to subtelomeric regions of short and long arms of pair 9 (Fig. 2).

### Main characteristics of *M. tiete* satellitome and comparison with catalogs of Colossomatinae

The satDNA catalog of *M. tiete* is composed of 32 families (Table 1). The repeat unit length varied between 24 and 2,265 bp, with a median of 121.5. We found a balance between short (< 100 bp) and long (> 100 bp) satDNAs, representing 46.8% and 53.2% of the catalog, respectively. The A + T-rich satDNAs were predominant, with content varying between 40 and 66.6% and a mean of 56.32%. Repeat landscape (**Supplementary Fig. 1**) is consistent with recent amplification dynamics.

**Table 1:** Main characteristics of *M. tiete* satellitome

SatDNA	RUL	A + T (%)	Abundance	Divergence
MtiSat01-650	650	40.1	0.004173716	2.75
MtiSat02-206	206	46.1	0.002758681	2.83
MtiSat03-24	24	66.6	0.00182858	15.31
MtiSat04-30	30	53.3	0.001756834	9.95
MtiSat05-42	42	54.76	0.001689457	13.36
MtiSat06-177	177	66.1	0.001369735	4.11
MtiSat07-2108	2108	61.2	0.001247475	9.56
MtiSat08-51	51	62.7	0.00116887	13.01
MtiSat09-54	54	53.7	0.001013885	12.16
MtiSat10-72	72	65.2	0.000960619	7.76
MtiSat11-74	74	58.1	0.000897454	11.77
MtiSat12-1119	1119	45.1	0.000876148	26.7
MtiSat13-66	66	62.1	0.000867789	7.2
MtiSat14-68	68	63.2	0.000831528	6.48
MtiSat15-101	101	59.4	0.000781993	5.36
MtiSat16-2265	2265	52	0.000586849	14.46
MtiSat17-1057	1057	48.5	0.000585767	1.38
MtiSat18-28	28	46.4	0.00047984	12.04
MtiSat19-221	221	60.1	0.00043594	8.73
MtiSat20-62	62	66.1	0.000414246	14.22
MtiSat21-946	946	62.4	0.000387092	2.55
MtiSat22-142	142	51.4	0.000373817	7.7
MtiSat23-1440	1440	59	0.000350487	3.51
MtiSat24-60	60	40	0.000345526	9.43
MtiSat25-1258	1258	56.5	0.000315714	10.48
MtiSat26-24	24	62.5	0.000230351	10.54
MtiSat27-34	34	55.8	0.000209212	8.46
MtiSat28-165	165	55.1	0.000160133	4.07
MtiSat29-1738	1738	53.5	0.000131676	1.74
MtiSat30-40	40	60	0.000119471	8.32
MtiSat31-1112	1112	54.1	0.000106657	4.9
MtiSat32-903	903	60.1	8.38E-05	6.75

We investigated the conservation patterns between the satDNA catalogs of *M. tiete* and those previously described catalogs for *C. macropomum* and *P. mesopotamicus* (Table 2). The comparative analysis revealed that 12 satDNAs of *M. tiete* are conserved in another catalog of Colossomatinae. Nine satDNAs of *M. tiete* are present in the two catalogs of Colossomatinae, with at least 80% similarity between the consensus sequences. MtiSat07-2108 and MtiSat27-34 were shared only between *M. tiete* and *P. mesopotamicus*, with 50.27% and 81.81% similarity, respectively. In addition, MtiSat08-51 was detected only in *the C. macropomum* satellitome, with 66.66% similarity. Interestingly, nine out of the twelve shared satDNAs of *M. tiete* are short, with less than 100 bp.

**Table 2:** Conserved satDNAs between *M. tiete*, *C. macropomum,* and *P. mesopotamicus*

*M. tiete*	*C. macropomum*	*P. mesopotamicus*
MtiSat04-30	CmaSat18-30	PmeSat19-30
MtiSat05-42	CmaSat09-42	PmeSat07-42
MtiSat06-177	CmaSat03-177	PmeSat08-177
MtiSat07-2108	-	PmeSat09-696
MtiSat08-51	CmaSat29-34	-
MtiSat09-54	CmaSat31-54	PmeSat21-54
MtiSat10-72	CmaSat20-72	PmeSat12-72
MtiSat13-66	CmaSat33-66	PmeSat18-67
MtiSat14-68	CmaSat22-68	PmeSat17-65
MtiSat15-101	CmaSat34-101	PmeSat27-102
MtiSat18-28	CmaSat21-28	PmeSat22-28
MtiSat27-34	-	PmeSat07-42

**Fig. 3:**
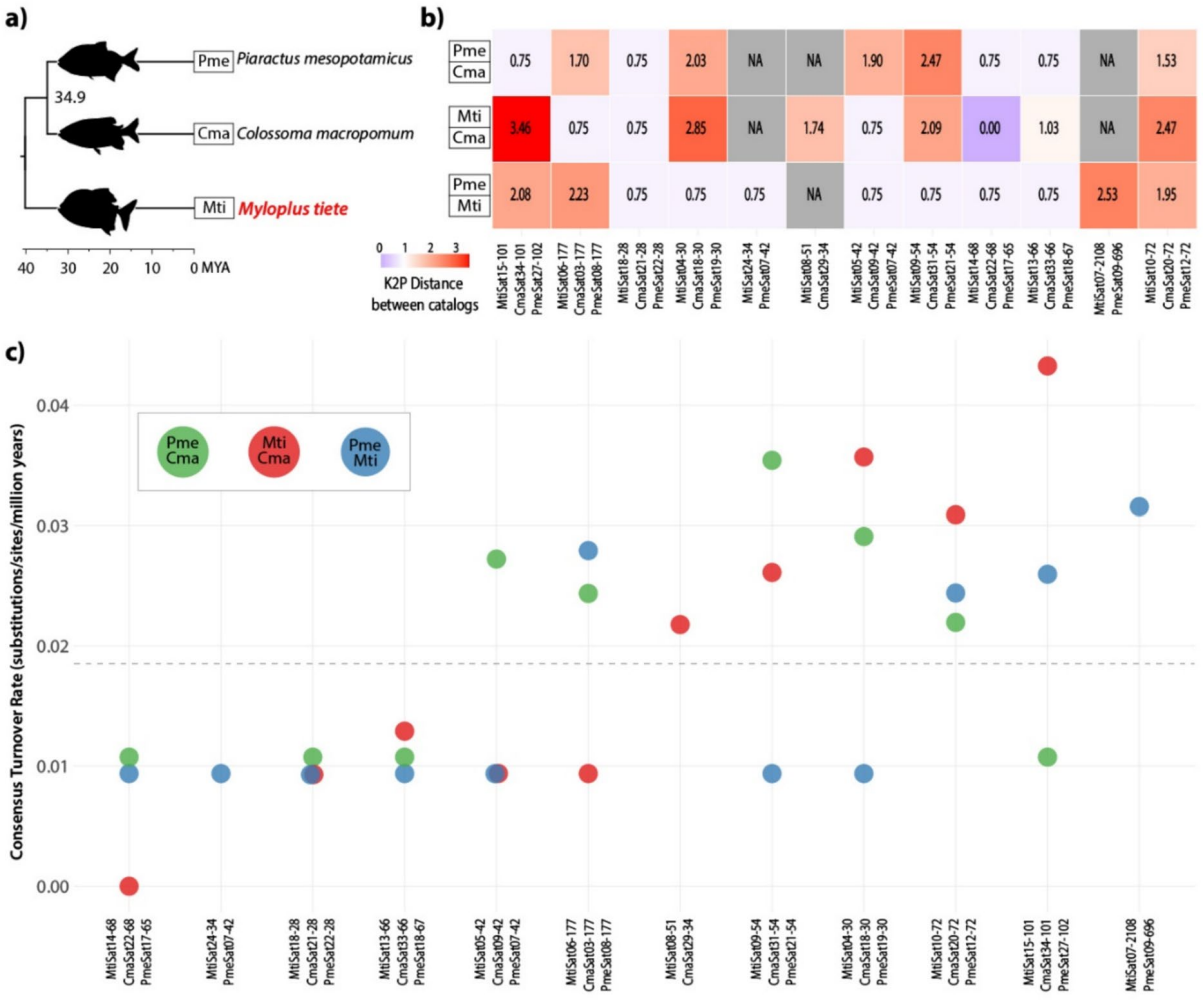
Summary of conserved satellite DNA families among Serrasalmidae. **a)** Time-calibrated phylogenetic tree of the compared species (*Piaractus mesopotamicus*, *Colossoma macropomum*, and *Myloplus tiete*) according to TimeTree5 (Kumar et al. 2022). The target species from the present study is highlighted in red. The divergence time between *P. mesopotamicus* and *C. macropomum* is indicated (34.9 Mya), while between *M. tiete* and others corresponds to the timescale (40 Mya). **b)** Heatmap of the Kimura 2-parameter genetic distance between conserved satDNAs among the three compared species. **c)** Consensus turnover rate (CTR) of the shared satDNA sequences in substitutions per site per million years. The dashed line indicates the mean value of CTR

To investigate the evolution of such shared satDNAs, we employed a comparison of genetic distances (K2P) and consensus turnover rates (CTR) based on the species divergence time, compiled from several time-calibrated phylogenies in TimeTree5 (Kumar et al. 2022), as summarized in Fig. 3. A mean of 1.419 K2P distance was observed across 30 pairwise comparisons between shared satDNAs (**Supplementary Table 2**). The consensus turnover rate (CTR) averaged 1.85e-8 substitutions/site/year (0.019 substitutions/site/million years), suggesting non-extreme evolutionary rates. Comparative analysis among species pairs showed that *M. tiete* versus *C. macropomum* exhibited the highest mean K2P distance (1.59 ± 1.11), followed by *C. macropomum* versus *P. mesopotamicus* (1.40 ± 0.67) and *M. tiete* versus *P. mesopotamicus* (1.28 ± 0.74). Consequently, CTR values showed corresponding patterns across these comparisons (0.0199 ± 0.0139, 0.0201 ± 0.0096, and 0.0160 ± 0.0093 substitutions/site/million years, respectively). ANOVA revealed no statistically significant differences in K2P distances among species comparisons (F = 0.347, p = 0.71), suggesting no detectable differences in divergence levels among these lineages. When comparing each shared satDNA family, they displayed considerable variation in evolutionary rates. The highest K2P distance was observed between MtiSat07-2108/PmeSat09-696 (2.53), consequently displaying the highest CTR (0.0316 substitutions/site/million years), while MtiSat14-68/CmaSat22-68/PmeSat17-65 showed the lowest values (K2P = 0.50, CTR = 0.0067 substitutions/site/million years). Notably, MtiSat18-28/CmaSat21-28/PmeSat22-28 demonstrated the highest conservation with zero standard deviation in K2P distances across all comparisons.

**Fig. 4:**
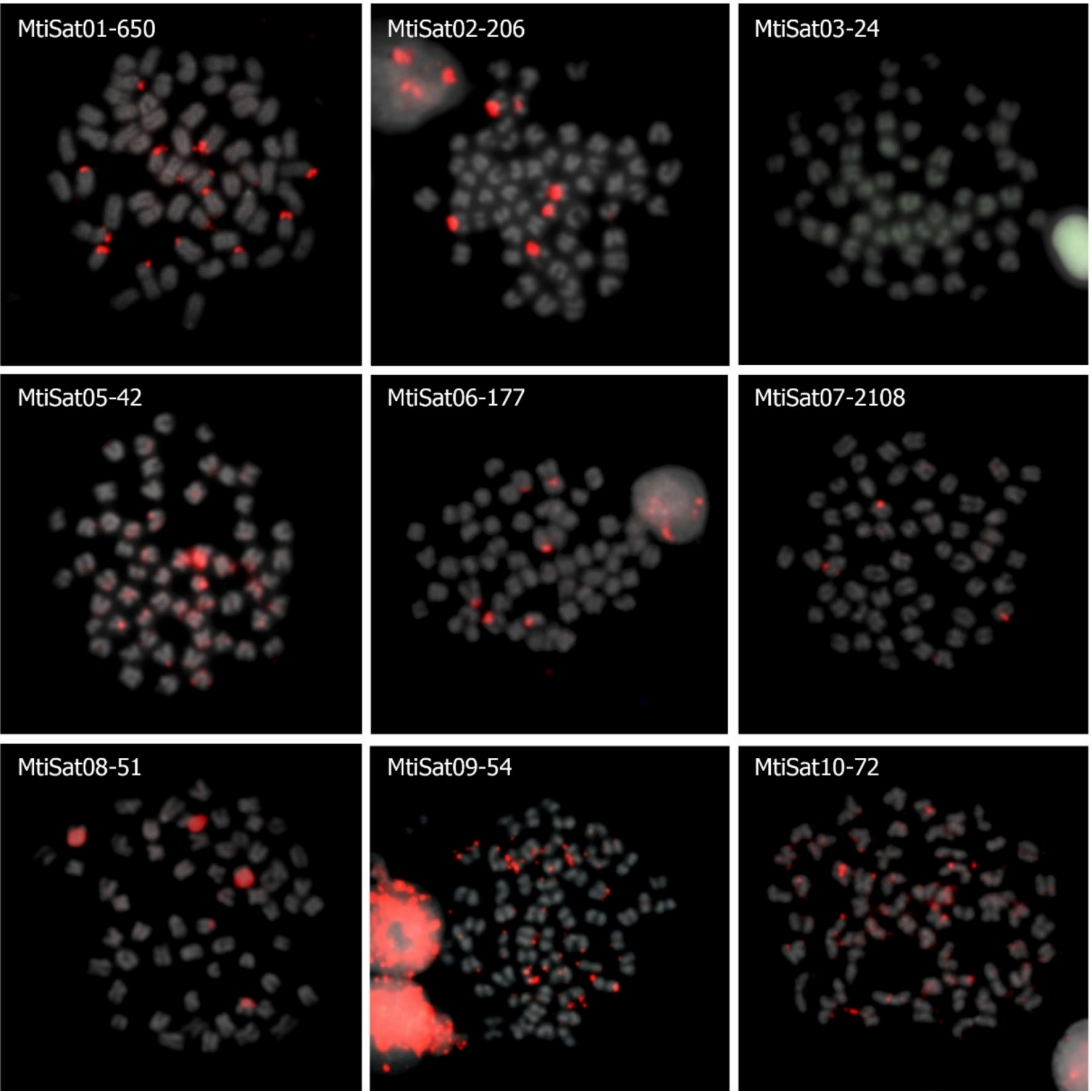
Metaphase plates of *M. tiete*, highlighting the chromosome location of the 10 most abundant satDNAs

We chose the ten most abundant satDNAs to perform cytogenetic mapping by FISH (Fig. 4). The only exception was MtiSat04-30, which failed in PCR amplification. In general, the selected satDNAs demonstrated clusterization in pericentromeric regions or subtelomeric regions, with only MtiSat03-24 considered non-clustered. Satellites MtiSat05-42, MtiSat09-54, and MtiSat10-72 present a dispersed pattern of distribution, especially in telomeric regions. We detected four satDNAs forming clusters in pericentromeric regions of six (MtiSat02-206, MtiSat06-177, and MtiSat08-51) and two chromosomes (MtiSat07-2108). Finally, MtiSat01-650 was the only one to present both subtelomeric and pericentromeric clusters in acrocentric chromosomes.

## Discussion

### The first karyotype for *Myloplus tiete*

The first cytogenetic characterization of *M. tiete* reveals a diploid number of 2n = 58 chromosomes, consistent with previous reports for other *Myloplus* species (Favarato et al. 2021) and reinforcing the chromosomal conservation of diploid numbers observed within the Myleinae subfamily. However, variations in karyotype macrostructure can be observed between the herein described *M. tiete* karyotype and other *Myloplus* species (Favarato et al. 2021). While *M. tiete* presents a karyotype formula of 16m + 20sm + 22a, other *Myloplus* species present a high variation in karyotype formula, including the complete absence of acrocentric elements in *M. lobatus* and *M. schomburgkii*, which leads to an increase in metacentric chromosomes in those species. Considering the phylogenetic relationship between *Myloplus* species (Mateussi et al. 2020), this can be attributed to apomorphic centric fusions that occurred independently in these lineages.

In this sense, the karyotype of *M. tiete* resembles more the karyotype of *M. asterias* and *M. rubripinnis*, composed of metacentric, submetacentric, and acrocentric chromosomes. Notably, *M. tiete* has two less metacentric pairs when compared to *M. asterias* and *M. rubripinnis* but shares the number of acrocentric pairs with *M. asterias* (Favarato et al. 2021). Since there is also an increase in submetacentric pairs in *M. tiete* (8 pairs in *M. asterias*, 9 pairs in *M. lobatus*, and 10 pairs in *M. tiete*), pericentric inversions might also play an important role in chromosome evolution in the genus, since the diploid number is conserved.

The heterochromatin distribution pattern observed in *M. tiete*, with blocks predominantly located in centromeric regions of acrocentric chromosomes and additional subtelomeric regions in chromosome pair 9, is consistent with the general organization of constitutive heterochromatin in most fish species (Bertollo et al. 2017). The presence of subtelomeric heterochromatin in specific chromosome pairs reflects the distribution of certain satDNA sequences, such as MtiSat01-650 (Fig. 4), although the presence of other repeats not explored here cannot be ruled out.

### Satellite DNA evolution in Serrasalmidae

The *M. tiete* satellitome presents standard characteristics for fish catalogs, such as the balanced distribution between short and long satDNAs (46.8% and 53.2%, respectively) and the predominance of AT-rich sequences (mean AT content = 56.32%), as also generally present in vertebrates (Garrido-Ramos 2017; Šatović-Vukšić and Plohl 2023). Notably, the comparative analysis with *C. macropomum* and *P. mesopotamicus* (Colossomatinae) satellitomes reveals long-term conservation of satDNA sequences across Serrasalmidae subfamilies, persisting since the Late Eocene (~ 40 Mya). Long-term conservation of satDNA sequences is also observed through several Characiformes lineages (dos Santos and Calegari et al. 2021), but also in other animal groups such as bivalves (Plohl et al. 2010) and birds (Peona et al. 2022).

The identification of 12 conserved satDNA families between *M. tiete* and Colossomatinae species, with nine sequences showing > 80% similarity, supports the library hypothesis of satDNA evolution, which states that related species share a common satDNA catalog with species-specific amplification events (Fry and Salser 1977). Such phenomenon is commonly observed in several lineages of fish (Utsunomia et al. 2017; dos Santos and Calegari et al. 2021; Sassi et al. 2025; Singchat et al. 2025), mammals (Ahmad et al. 2020; Gutiérrez et al. 2023; Dudka et al. 2025), reptiles (Lisachov et al. 2023; Lisachova et al. 2025), birds (Kretschmer et al. 2024; Setti et al. 2024; Pozzobon et al. 2025), amphibians (Souza et al. 2025; Vidal et al. 2025a), insects (Mestrović et al. 1998; Palacios-Gimenez et al. 2020; Vidal et al. 2025b), mollusks (Šatović et al. 2018; Castro et al. 2026), and plants (Senderowicz et al. 2025; del Bosque et al. 2014). On the other hand, the conservation of short satDNA sequences (< 100 bp) is especially noteworthy, as these elements are typically subject to higher rates of concerted evolution due to their clustered organization (Plohl et al. 2012; Garrido-Ramos 2017).

Indeed, the conservation of these satDNAs over 40 Myr, combined with the absence of significant differences in K2P distances among species comparisons (F = 0.347, p = 0.71), is consistent with the idea that concerted evolution may have operated at moderately similar rates across Serrasalmidae lineages (mean CTR = 0.019 substitutions/site/million years). Concerted evolution proceeds through recursive cycles of amplification and degeneration driven by unequal crossing over and gene conversion, processes that simultaneously homogenize repeats within genomes and accelerate divergence between isolated populations (Dover 1982; Elder and Turner 1995; Ugarković and Plohl 2002). The moderate to slow turnover rate observed here is consistent with the hypothesis that long generation times of these large-bodied species could potentially contribute to slower mutation fixation rates (Goes et al. 2023). While this interpretation is supported by life-history expectations, additional comparative data across lineages would help to further evaluate its generality. Notably, the extreme conservation of specific families, particularly MtiSat18‑28/CmaSat21‑28/PmeSat22‑28, which exhibits no detectable divergence (zero standard deviation in K2P distances across all comparisons) across ~ 40 million years, suggests that concerted evolution may not act uniformly across the satDNA catalogs of this order. Indeed, the marked variation in turnover rates among all other orthologous families in Serrasalmidae (ranging from 0.0067 to 0.0316 substitutions/site/Myr – Table 2) mirrors observations in other taxa where the efficiency of concerted evolution varies among satDNA families (e.g., Camacho et al. 2022).

The maintenance of such sequences over extended evolutionary periods may indicate that specific biological or structural constraints limit their divergence. Mechanistically, the conservation of these specific short and A-T-rich satDNA may reflect selection acting upon their physical structure rather than solely their nucleotide sequence. Evidence from two related mouse species (Dudka et al. 2025) demonstrates that shared pericentromeric AT-rich satellites are not merely byproducts of replication, as their DNA structure is recognized by essential proteins for proper pericentromere packing and rigidity during meiosis, and the disruption of such a mechanism leads to severe satDNA stretching and chromosomal instability. Although the pericentromeric A + T-rich sequences CmaSat05 and PmeSat05 of *C. macropomum* and *P. mesopotamicus* (Goes et al. 2023) have no orthologous in *M. tiete*, sequences that are present in pericentromeric clusters in *M. tiete* are also A + T-rich (MtiSat06-177, MtiSat07-2108, MtiSat08-51, Table 1, Fig. 4) and have orthologous in the related Serrasalmidae species (Table 2, Fig. 3). Notably, only the orthologous of MtiSat06-177 (CmaSat03-177) was FISH mapped in *C. macropomum* (Goes et al. 2023), showing terminal signals at both arms of three pairs and on long arms of three other pairs, mainly located on heterochromatic regions. We therefore suggest that the conserved AT-rich satDNAs in Serrasalmidae may persist due to intrinsic structural properties (e.g., DNA curvature and minor groove width) that may be functionally optimal for the recruitment of heterochromatin factors, thereby exerting stabilizing selective pressure against divergence. This structural constraint, combined with the proposed reduced fixation rates of mutations due to the long generation times of these large-bodied species (Goes et al. 2023), may help explain the moderated consensus turnover rates observed for the shared sequences. Clearly, experimental characterization of protein-binding profiles and meiotic chromosome behavior in characiform models will be required to test whether these conserved satellites are functional analogs of mammalian pericentromeric repeats.

In conclusion, the first cytogenetic characterization of *Myloplus tiete* reveals a conserved diploid number but with lineage-specific macrostructural rearrangements in the karyotype, aligned with previous trends for Serrasalmidae fish. The satellitome presents 12 sequences conserved across Serrasalmidae since the Late Eocene (~ 40 Mya), with overall moderate turnover rates and non-significant genetic distances among the subfamilies Myleinae and Colossomatinae. Marked variation among orthologous families, including one with full conservation of sequence, may indicate that structural constraints could influence the heterogeneity of concerted evolution among Serrasalmidae satDNAs. Further studies focusing on protein-DNA interactions will be necessary to determine whether this putative association reflects functional constraint or neutral evolutionary persistence.

## Supplementary Information

Below is the link to the electronic supplementary material.

Supplementary file1 (DOCX 187 KB)

Supplementary file2 (DOCX 16 KB)

Supplementary file3 (DOCX 17 KB)

## Data Availability

All data supporting the findings of this study are available within the paper and its Supplementary Information. The raw reads were deposited in the Sequence Read Archive (SRA-NCBI) under accession number SRR37277025. The satellitome is deposited in NCBI under accession numbers PZ024035—PZ024066.
