# Supplementary figures and images for: Cytogenomics of Myloplus tiete reveals conserved satellite DNAs since the Late Eocene in Serrasalmidae (Teleostei, Characiformes)
Source: Chromosome Res. 2026 May 8;34(1):10. doi: 10.1007/s10577-026-09801-w (PMC13156159; doi:10.1007/s10577-026-09801-w)

**
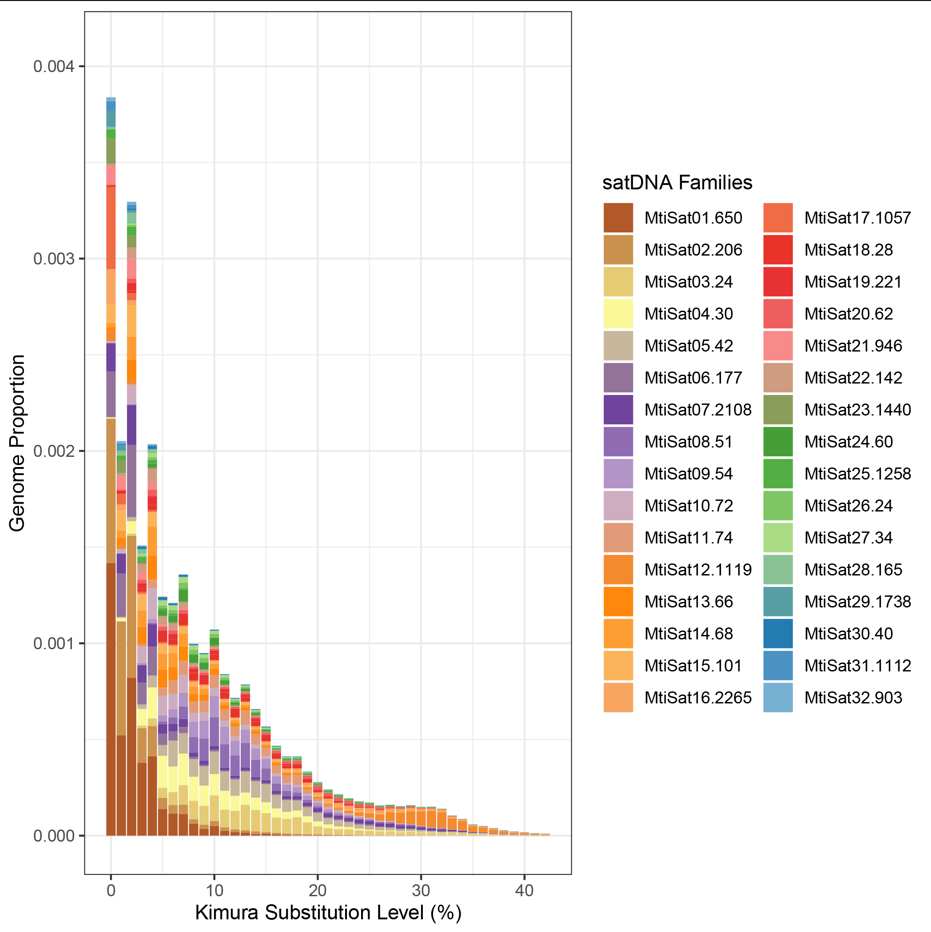
**

**Supplementary Figure 1.** Repeat landscape for the satDNA catalog of *Myloplus tiete*.

Supplement: Supplementary file 1 — Supplementary file1 (DOCX 187 KB) [file 10577_2026_9801_MOESM1_ESM.docx]
