# Supplementary material for: Cytogenomics of Myloplus tiete reveals conserved satellite DNAs since the Late Eocene in Serrasalmidae (Teleostei, Characiformes)
Source: Chromosome Res. 2026 May 8;34(1):10. doi: 10.1007/s10577-026-09801-w (PMC13156159; doi:10.1007/s10577-026-09801-w)
Supplement: Supplementary file 2 — Supplementary file2 (DOCX 16 KB) [file 10577_2026_9801_MOESM2_ESM.docx]

**TABLE SX.** Satellite DNA primers used by FISH mapping.

| **Satellite** | **Primer F** | **Primer R** |
| --- | --- | --- |
| MtiSat01-650 | GGTTCAGGTGTGCTTCGG | GATACGCCCTCGGATTCC |
| MtiSat02-206 | CCTCGGGTAGACTTTTGGC | GAAAGTGCGCCGTGGTCG |
| MtiSat05-42 | AGCACAAGGTAGGTGTTTCT | TCCACTCACTGGCCACTTT |
| MtiSat06-177 | TTTGATGCCTTAGAGTGAATA | AGTTGTTGGAAAATGCCTG |
| MtiSat07-2108 | ACGTATTGCACAGCCCAAAC | TGCCATGTATTTGTAGACCC |
| MtiSat08-51 | ACCTTAAAAGCAGTTCCACC | GGAATTGCACTTAGGTAACAC |
| MtiSat09-54 | ACATCAAATAAACAAACGTCGC | TTGTTAATGACGCCTTTAGAGG |
| MtiSat10-72 | TGTTCTACAGTTTCTCATTAG | GACTCAGTTTATATCACAATAC |
