# Supplementary material for: Cytogenomics of Myloplus tiete reveals conserved satellite DNAs since the Late Eocene in Serrasalmidae (Teleostei, Characiformes)
Source: Chromosome Res. 2026 May 8;34(1):10. doi: 10.1007/s10577-026-09801-w (PMC13156159; doi:10.1007/s10577-026-09801-w)
Supplement: Supplementary file 3 — Supplementary file3 (DOCX 17 KB) [file 10577_2026_9801_MOESM3_ESM.docx]

**Supplementary Table 2.** Evolutionary analysis among conserved satDNAs in Serrasalmidae, representing the Kimura 2-parameter genetic distance (k2p) and consensus turnover rate (CTR) mean values and standard deviation (SD).

| **Shared SatDNA** | **Length (bp)** | **Comparisons** | **Mean k2p** | **SD k2p** | **Mean CTR** | **SD CTR** |
| --- | --- | --- | --- | --- | --- | --- |
| MtiSat15-101  CmaSat34-101  PmeSat27 | 101/102 | 3 | 2.10 | 1.36 | 0.0267 | 0.0163 |
| MtiSat06-177  CmaSat03-177  PmeSat08-177 | 177 | 3 | 1.56 | 0.752 | 0.0206 | 0.00985 |
| MtiSat18-28  CmaSat21-28  PmeSat22-28 | 28 | 3 | 0.75 | 0 | 0.00983 | 0.000791 |
| MtiSat04-30  CmaSat18-30  PmeSat19-30 | 30 | 3 | 1.88 | 1.06 | 0.0247 | 0.0137 |
| MtiSat21-34  PmeSat07-42 | 34/42 | 1 | 0.75 | NA | 0.00938 | NA |
| MtiSat08-51  CmaSat29-34 | 34/51 | 1 | 1.74 | NA | 0.0218 | NA |
| MtiSat05-42  CmaSat09-42  PmeSat07-42 | 42 | 3 | 1.13 | 0.664 | 0.0153 | 0.0103 |
| MtiSat09-54  CmaSat31-54  PmeSat21-54 | 54 | 3 | 1.77 | 0.904 | 0.0236 | 0.0132 |
| MtiSat14-68  CmaSat22-68  PmeSat17-65 | 65/68 | 3 | 0.50 | 0.433 | 0.00671 | 0.00585 |
| MtiSat13-66  CmaSat33-66  PmeSat18-67 | 66/67 | 3 | 0.844 | 0.163 | 0.0110 | 0.00178 |
| MtiSat07-2108  PmeSat09-696 | 696/2108 | 1 | 2.53 | NA | 0.0316 | NA |
| MtiSat10-72  CmaSat20-72  PmeSat12-72 | 72 | 3 | 1.99 | 0.471 | 0.0258 | 0.00462 |
